# Hypoxia as a Central Regulator of Plasma Membrane Phosphohydrolase Enzymes: Possible Roles in Extracellular Phosphate Generation and Adenosine Metabolism

**DOI:** 10.3390/membranes15120381

**Published:** 2025-12-15

**Authors:** Pedro Henrique Silva de Oliveira, Beatriz Bereda Silva-Freitas, José Roberto Meyer-Fernandes, Marco Antonio Lacerda-Abreu

**Affiliations:** 1 Instituto de Bioquímica Médica Leopoldo de Meis, Centro de Ciências da Saúde, Universidade Federal do Rio de Janeiro, Rio de Janeiro 21941-590, RJ, Brazil; pholiveirac18@gmail.com (P.H.S.d.O.); beatrizberedaf@gmail.com (B.B.S.-F.); 2 Departamento de Bioquímica, Instituto de Biologia Roberto Alcantara Gomes (IBRAG), Universidade Do Estado Do Rio de Janeiro, Rio de Janeiro 20550-013, RJ, Brazil

**Keywords:** hypoxia-induced metabolic reprogramming, ectophosphohydrolase regulation, adenosine and phosphate metabolism in cancer

## Abstract

This article presents a conceptual perspective proposing that hypoxia acts as a unifying regulator of plasma membrane phosphohydrolases. We propose that oxygen sensing at the cell surface integrates adenosine and phosphate metabolism to sustain tumour adaptation. Within the oxygen- and nutrient-deprived tumour microenvironment, inorganic phosphate (Pi) and adenosine function as metabolic substrates and signalling mediators that promote cell proliferation, survival, and immune evasion. Stabilisation of hypoxia-inducible factor-1α (HIF-1α) enhances the expression and catalytic activity of specific phosphohydrolases, notably the ectonucleotidases CD39 (NTPDase1) and CD73 (ecto-5′-nucleotidase), which drive adenosine accumulation and immunosuppression. Conversely, the activity of transmembrane prostatic acid phosphatase (TM-PAP), responsible for hydrolysing phosphate esters such as p-nitrophenylphosphate (pNPP) and AMP, is inhibited under hypoxia through oxidative and kinase-dependent mechanisms. Collectively, these mechanisms characterise the plasma membrane as a dynamic metabolic interface, where oxygen sensing coordinates adenosine and phosphate turnover, thereby promoting tumour adaptation across hypoxic environments. We propose that hypoxia orchestrates a dual regulatory loop connecting adenosine accumulation and phosphate turnover at the tumour cell surface, providing a conceptual basis for future mechanistic studies.

## 1. Introduction

Hypoxia frequently develops within solid tumours as a consequence of uncontrolled cell proliferation and inadequate vascular perfusion [1]. It represents a defining characteristic of the tumour microenvironment and has been associated with metabolic reprogramming, angiogenesis, invasion, and therapeutic resistance [2,3]. Persistent hypoxic signalling enables tumour cells to withstand metabolic stress and to acquire more aggressive phenotypes [2]. Despite extensive progress in the field, the complex nature of these signalling networks still limits a comprehensive understanding of their molecular mechanisms and hinders the development of effective therapeutic strategies [1]. As oxygen availability decreases, extracellular nucleotide metabolism is markedly affected, altering the catalytic activity of plasma membrane-associated enzymes responsible for the regulation of purinergic signalling within the tumour microenvironment. In this context, HIF-1α stabilisation may also occur through ROS-dependent mechanisms associated with enhanced oxidative metabolism, contributing to pseudo-hypoxic signalling and variability in tumour responses [4].

The tumour microenvironment (TME) constitutes a decisive component in cancer development and progression [5]. In hypoxic regions, it represents a dynamic interface where tumour, stromal, and immune cells interact under continuous metabolic and mechanical constraints. In this context, purinergic signalling assumes a regulatory role largely mediated by extracellular adenosine triphosphate (ATP), which is released by virtually all cell types in response to both physiological and pathological stimuli [6]. Under normal physiological conditions, extracellular ATP levels remain within the nanomolar range [7]; however, within the TME, these concentrations may reach several hundred micromolar, reflecting substantial metabolic and structural remodelling during tumour progression [6,7].

The homeostasis of extracellular ATP is strictly controlled by plasma membrane-bound ectonucleotidases and ectophosphatases, collectively designated ectophosphohydrolases, which display distinct substrate specificities [5]. These enzymes include (a) ectonucleoside triphosphate diphosphohydrolases (ENTPDases), responsible for the hydrolysis of nucleotide triphosphates (NTPs) and diphosphates (NDPs); (b) ectonucleotide pyrophosphatase/phosphodiesterases (E-NPPs), which catalyse NTP hydrolysis and, in the case of NPP2, dephosphorylate ATP, ADP, AMP, and PPi; (c) ecto-5′-nucleotidase (E-5NT), which converts nucleotide monophosphates (NMPs) into nucleosides; (d) alkaline phosphatases (ALPs), which act on a wide range of phosphorylated substrates, including NTPs, NDPs, NMPs, and PPi; and (e) acid ectophosphatases, which hydrolyse organic phosphomonoesters and phosphoproteins [5,8,9].

Although the roles of purinergic signalling and hypoxia-driven metabolic adaptation have been extensively examined, the molecular interconnection between oxygen deprivation and the regulation of surface phosphohydrolases remains poorly elucidated. These enzymes operate as metabolic sensors that couple extracellular phosphate and nucleotide turnover to the cellular response under hypoxic stress, thereby promoting tumour adaptation and immune modulation. Here, we advance a unifying hypothesis proposing that hypoxia-driven modulation of plasma membrane phosphohydrolases establishes a regulatory loop linking extracellular phosphate release to adenosine accumulation. This mechanistic interpretation provides insight into how oxygen availability influences extracellular metabolism and tumour adaptation. We acknowledge that baseline ectoenzyme expression, tissue-specific phosphate metabolism, stromal composition, immune infiltrate, and hypoxia kinetics differ substantially across tumour types. To illustrate this conceptual model, we summarise evidence from different cancer types in which hypoxia-driven phosphohydrolase activity contributes to adenosine accumulation and phosphate dynamics.

## 2. Non-Small Lung Cancer

Lung tumours represent one of the most hypoxic types of human malignancies, where oxygen deprivation profoundly reprogrammes both metabolic and immune dynamics. In non-small cell lung cancer (NSCLC), hypoxia activates adaptive molecular pathways that promote cell survival, stemness, and resistance to therapeutic interventions [10]. These effects are mainly orchestrated by the transcription factor HIF-1α, which regulates a broad set of genes involved in angiogenesis, metabolic reorganisation, and immune evasion [10].

**Figure 1:**
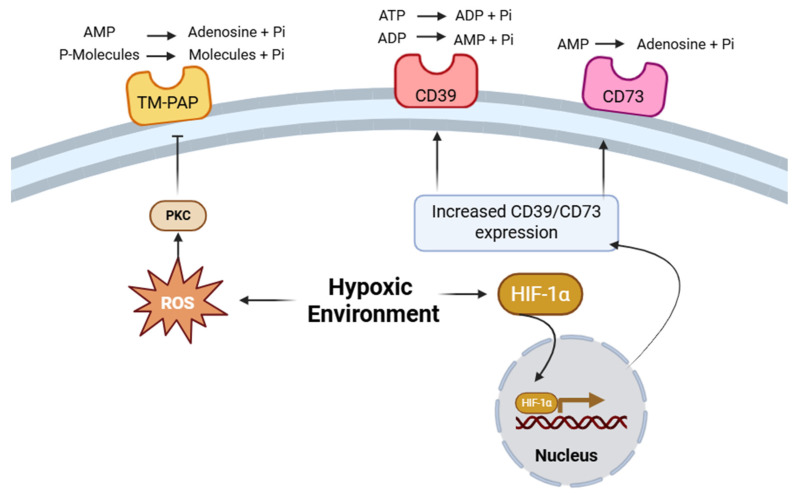
Schematic representation of the proposed hypoxia-driven regulatory loop integrating adenosine and phosphate metabolism at the tumour cell surface. Hypoxia stabilises HIF-1α, which enhances CD39/CD73 expression and adenosine generation, while reactive oxygen species (ROS) and PKC-dependent signalling inhibit TM-PAP, thereby modulating phosphate turnover. This integrated regulation exemplifies the proposed model in which oxygen sensing coordinates extracellular metabolism and supports tumour adaptation.

In this context, it has been demonstrated that hypoxia significantly enhances the immunosuppressive properties of myeloid-derived suppressor cells (MDSCs) through HIF-1α-dependent upregulation of the ectonucleotidases CD39 and CD73 [11]. By employing peripheral blood and tumour specimens from patients with NSCLC, in parallel with in vitro assays using MDSCs and A549 lung adenocarcinoma cells, the authors observed that both chemical (CoCl_2_) and physiological (1.5% O_2_) hypoxia strongly induced CD39 and CD73 expression (Figure 1) [11]. Pharmacological inhibition of HIF-1α with MeoE_2_ suppressed this effect (Table 1).

Complementing these immunological observations, another study provided additional evidence for the metabolic component of adenosinergic regulation in NSCLC [12]. Exposure of A549 and H1299 cells to 1% O_2_ for 48 h resulted in a marked increase in CD73 mRNA and protein expression, particularly in A549 cells, which was accompanied by the upregulation of lactate dehydrogenase A (LDHA), a key enzyme driving anaerobic glycolysis (Table 1) [12]. Activation of LDHA enhanced glycolytic ATP production, part of which was released into the extracellular milieu, serving as a substrate for CD73-mediated AMP hydrolysis and subsequent adenosine generation [12].

**Figure 2:**
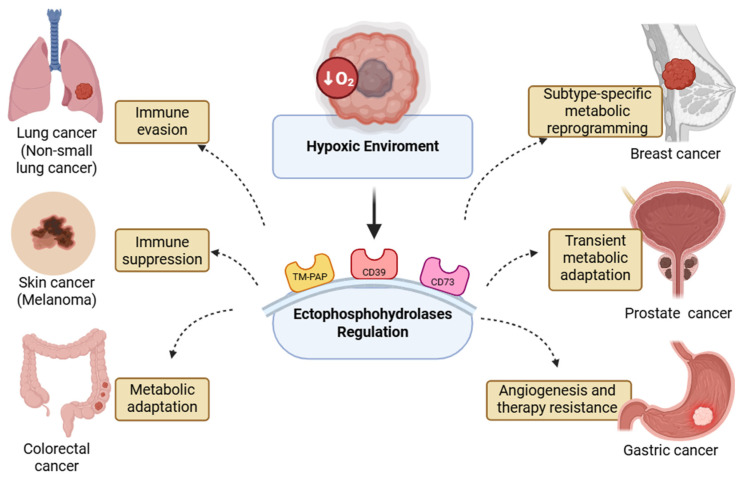
Conceptual overview of the proposed hypoxia-driven regulatory loop across tumour types. This integrative model links HIF-1α activation to adenosine accumulation and phosphate modulation, illustrating a unifying mechanism of tumour adaptation.

Taken together, these results support the perspective that HIF-1α-driven induction of ectonucleotidases and LDHA-mediated glycolytic adaptation may represent interlinked processes within a hypoxia-regulated circuit. Such coordination between adenosine metabolism and energy reprogramming could underlie the establishment of an immunosuppressive, therapy-resistant tumour microenvironment (Figure 2).

## 3. Colorectal Cancer

Colorectal cancer represents one of the most common and lethal malignancies worldwide and is distinguished by pronounced tumour heterogeneity and remarkable metabolic adaptability. Within its microenvironment, hypoxia constitutes a major determinant of malignant progression, promoting epithelial-to-mesenchymal transition, angiogenesis, and immune evasion [20,21]. In metastatic colorectal cancer (mCRC), the hypoxic milieu profoundly alters adenosine metabolism, thereby influencing disease progression and modulating therapeutic responsiveness (Figure 2) [13].

Accumulating molecular evidence indicates that HIF-1α transcriptionally regulates a subset of plasma membrane ectophosphohydrolases, including CD39 and CD73, which catalyse the sequential hydrolysis of extracellular ATP into adenosine, concomitantly releasing Pi [13] (Figure 1). The resultant accumulation of adenosine exerts dual effects: it suppresses cytotoxic lymphocyte activity and promotes endothelial proliferation, collectively favouring a pro-angiogenic and immunosuppressive microenvironment (Table 1) [13].

In addition to these mechanistic insights, a pharmacogenomic analysis encompassing 451 patients with mCRC enrolled in the FIRE-3 and TRIBE clinical trials revealed that single-nucleotide polymorphisms (SNPs) in genes associated with adenosine metabolism correlate with treatment outcome [13]. The CD39 rs11188513 C allele was linked to reduced overall and progression-free survival among patients receiving bevacizumab, implicating enhanced adenosinergic activity in resistance to VEGF blockade. Conversely, the CD73 rs2229523 A allele and the A2BR rs2015353 T/T genotype were associated with improved prognosis, suggesting that genetic variation within this pathway modulates vascular and immune responses [13]. Interestingly, the prognostic trend was inverted in cohorts treated with cetuximab, indicating that adenosine-related polymorphisms may influence therapeutic efficacy according to the predominant signalling axis engaged [13].

## 4. Breast Cancer

In aggressive breast tumours, such as triple-negative breast cancer (TNBC), oxygen deprivation amplifies adenosinergic signalling, thereby sustaining proliferation, motility, and immune evasion through the coordinated activity of CD39, CD73, and additional phosphohydrolases [14]. Within this heterogeneous disease, hypoxia induces subtype-specific metabolic and enzymatic adaptations that modulate extracellular nucleotide turnover and reconfigure immune escape mechanisms [14,22].

Under hypoxic conditions (1–5% O_2_), a significant upregulation of CD73 expression has been observed in both murine (4T1) and human (MDA-MB-231) cells, concomitant with increased HIF-1α transcriptional activity, suggesting a cooperative regulatory interaction [14]. Enhanced CD73 expression under hypoxia is associated with increased cell viability, whereas pharmacological inhibition with APCP or gene silencing attenuates this effect (Table 1) [14]. Although hypoxia alone does not substantially modify basal migratory capacity, inhibition of CD73 significantly reduces motility under both normoxic and hypoxic conditions. Furthermore, orthotopic implantation of 4T1 cells confirmed that CD73 silencing diminishes tumour growth, expression of EMT markers, and pulmonary metastasis [14].

Consistent with these findings, further evidence indicates that hypoxia preferentially amplifies adenosinergic signalling in more aggressive phenotypes [15]. When non-metastatic BT-474 (luminal A) and highly metastatic MDA-MB-231 (triple-negative) cells were exposed to 25 µM CoCl_2_ for 24 h, expression of CD39 and CD73 increased markedly, exhibiting a more pronounced induction in MDA-MB-231 cells (1.93-fold and 13.37-fold, respectively) compared with BT-474 cells (1.66-fold and 1.60-fold, respectively) (Table 1) [15]. This coordinated upregulation is associated with accelerated ATP hydrolysis and extracellular adenosine accumulation, contributing to immunosuppressive features within the tumour microenvironment [15].

Beyond the regulation of ectonucleotidases, hypoxia also affects the catalytic activity of surface phosphohydrolases involved in the control of extracellular phosphate homeostasis [16]. In luminal A breast cancer cells (MCF-7), short-term hypoxia (5% O_2_ for 1 h) results in a marked reduction in transmembrane prostatic acid phosphatase (TM-PAP) activity (Table 1) [16]. This enzyme catalyses the hydrolysis of phosphate esters, such as p-nitrophenylphosphate (pNPP) and AMP, thereby regulating phosphate and adenosine availability within the tumour microenvironment. Under hypoxic stress, ectophosphatase activity decreases significantly without impairing cell viability. This effect has been attributed to hydrogen peroxide (H_2_O_2_) accumulation, which oxidises and inhibits TM-PAP. The use of reactive oxygen species (ROS) scavengers restores enzyme activity, confirming oxidative inhibition as the primary regulatory mechanism. Moreover, activation of protein kinase C (PKC) further modulates TM-PAP through phosphorylation-dependent suppression (Figure 1) [16].

Collectively, these findings can be interpreted as evidence that hypoxia orchestrates the complementary regulation of ectonucleotidases and ectophosphatases in breast cancer, aligning adenosine and phosphate metabolism with the adaptive demands of tumour cells. Reduced oxygen availability appears to enhance adenosine production via CD39 and CD73 in aggressive subtypes, while transiently suppressing TM-PAP activity in luminal cells. This coordinated regulation exemplifies the proposed model in which hypoxia acts as a central determinant linking metabolic plasticity, immune modulation, and signalling reprogramming across breast cancer subtypes.

## 5. Prostate Cancer

Prostate tumours arise within fluctuating oxygen gradients that exert a profound influence on their purine metabolism and enzymatic profile [17,23]. Under hypoxic conditions, stabilisation of hypoxia-inducible factors triggers compensatory pathways that tightly regulate extracellular nucleotide turnover, prominently involving the ectonucleotidase CD73 (Figure 1). This enzyme establishes a functional association between oxygen sensing and increased extracellular adenosine availability [17,23].

Consistent with findings reported in breast tumours, hypoxia also modulates purinergic enzyme activity in prostate carcinoma, supporting its role as a universal regulator of surface phosphohydrolases [17]. In PC3 prostate cancer cells, acute hypoxia (1% O_2_ for 24 h) markedly enhances both the expression and catalytic activity of CD73, the enzyme responsible for the hydrolysis of extracellular AMP to adenosine. This regulatory effect appears to be selective, as other ectoenzymes involved in nucleotide metabolism, including NPP1 and adenosine deaminase (ADA), remain unaffected. Upon reoxygenation, CD73 expression and activity return to basal levels, revealing a reversible and oxygen-dependent regulatory mechanism that dynamically adjusts extracellular adenosine availability (Table 1 and Figure 2) [17].

In xenograft models, hypoxia-induced adenosine accumulation predominantly contributes to the early adaptive phase of tumour establishment, with minimal influence on subsequent tumour expansion. This transient response highlights the association between CD73-mediated modulation of extracellular metabolism and early hypoxic adaptation [17].

## 6. Melanoma

Among cutaneous malignancies, melanoma displays an extraordinary ability to adapt to hypoxic stress [24]. Oxygen deprivation sustains angiogenic and metabolic reprogramming while simultaneously remodelling the immune microenvironment through enhanced adenosine generation mediated by the ectonucleotidases CD39 and CD73. The accumulation of adenosine establishes an immunosuppressive niche that favours tumour persistence and progression (Figure 2) [18].

Recent findings indicate that hypoxia not only regulates ectoenzyme expression in tumour cells but also profoundly modulates their expression within immune compartments. In CD8^+^ T lymphocytes, exposure to low oxygen tension (~1.5% O_2_) markedly increases CD39 expression via HIF-1α-dependent transcriptional activation (Table 1) [18]. Deletion of HIF-1α completely abolishes this induction, confirming its direct regulatory role (Figure 1), whereas pharmacological alleviation of hypoxia reduces CD39 expression and restores T-cell effector activity. The subsequent reactivation of antitumour immunity enhances responsiveness to immunotherapeutic intervention [18]. It should be noted that, in melanoma, this evidence is currently supported by a single experimental study.

## 7. Gastric Cancer

The hypoxic microenvironment characteristic of gastric tumours serves as a potent inducer of metabolic and transcriptional adaptation [25]. Exposure of gastric cancer cell lines (BGC-823, HGC-27, and SGC-7901) to hypoxia (1% O_2_) or chemical hypoxia induced by CoCl_2_ markedly increases CD73 (ecto-5′-nucleotidase) expression through HIF-1α-dependent transcriptional activation (Table 1) [19]. Chromatin immunoprecipitation assays have demonstrated that HIF-1α directly binds to the promoter region of the CD73 gene, establishing a mechanistic link between oxygen sensing and adenosine metabolism [19] (Figure 1).

Functionally, CD73 activity sustains the Warburg phenotype by promoting glycolytic ATP release and extracellular adenosine formation, two complementary mechanisms that collectively enhance angiogenesis, immune evasion, and metabolic plasticity in oxygen-limited conditions. This coordinated response defines HIF-1α-driven CD73 induction as a pivotal mechanism underlying hypoxic adaptation and therapeutic resistance in gastric cancer [19]. At present, this conclusion is based on a single experimental study.

## 8. Conclusions

In this perspective, we propose that hypoxia orchestrates a dual regulatory axis controlling adenosine and phosphate metabolism through the modulation of plasma membrane phosphohydrolases. This integrated mechanism delineates a regulatory loop that sustains tumour cell adaptation under oxygen limitation. Across solid tumours, hypoxia emerges as a central determinant of extracellular metabolic reprogramming, modulating the activity of plasma membrane-associated phosphohydrolases that maintain adenosine and phosphate homeostasis. Under low oxygen tension, HIF-1α-driven upregulation of the ectonucleotidases CD39 and CD73 promotes adenosine accumulation, establishing an immunosuppressive and pro-survival microenvironment, while oxidative inhibition of transmembrane prostatic acid phosphatase (TM-PAP) alters phosphate dynamics at the cell surface.

Collectively, these observations support a conceptual model in which this response is empirically linked to hypoxia, whereas phosphatase-dependent modulation of extracellular phosphate remains a partially supported and still speculative dimension. This conceptual framework positions ectophosphohydrolases as candidate mediators of hypoxic adaptation and as potential metabolic biomarkers and therapeutic targets in cancer. Future investigations should explore the temporal coordination between CD39/CD73 induction and TM-PAP inhibition during hypoxic–reoxygenation cycles, considering tumour-specific differences in baseline ectoenzyme expression, phosphate handling, immune composition, and hypoxia dynamics.

## Figures and Tables

**Table 1:** Hypoxia-induced regulation of ectophosphohydrolases across cancer types.

Cancer Type	Model	Ectoenzyme	Hypoxia Conditions	Main Findings	Reference
Non-small cell lung cancer (NSCLC)	Patient blood and tumour samples; MDSCs and A549 cells	CD39, CD73	CoCl_2_; 1.5% O_2_	HIF-1α activation upregulated CD39/CD73 on MDSCs, enhancing immunosuppression and chemoresistance independently of mTOR.	[11]
	A549 and H1299 cells	CD73	1% O_2_, 48 h	Hypoxia increased CD73 and LDHA, reinforcing adenosine production and immune evasion.	[12]
Colorectal cancer	FIRE-3 and TRIBE clinical trials (*n* = 451 patients)	CD39, CD73	Not experimentally induced	CD39/CD73 polymorphisms correlated with survival under bevacizumab; the HIF-1α–adenosine pathway is linked to VEGF-blockade resistance.	[13]
Breast cancer	4T1 and MDA-MB-231 cells; BALB/c mice	CD73	1–5% O_2_	Hypoxia elevated CD73 expression and EMT; CD73 inhibition reduced migration, viability, and lung metastases.	[14]
	BT-474 and MDA-MB-231 cells	CD39, CD73	CoCl_2_ (25 µM, 24 h)	Hypoxia upregulated CD39/CD73, predominantly in TNBC, promoting immune evasion.	[15]
	MCF-7 cells	TM-PAP	5% O_2_, 1 h	Hypoxia reduced ectophosphatase activity (pNPP and AMP hydrolysis) via H_2_O_2_ generation and PKC activation.	[16]
Prostate cancer	PC3 cells; xenografts in SCID mice	CD73	1% O_2_ (≤24 h); reoxygenation 1 h	Hypoxia doubled CD73 activity and AMP hydrolysis; NPP1 and ADA were unchanged; high ecto5′nucleotidase activity occurred in hypoxic xenografts.	[17]
Melanoma	B16-F10 murine model; T cells in vitro	CD39	1.5% O_2_	HIF-1α-dependent CD39 expression on exhausted CD8^+^ T cells enhanced immunosuppression; hypoxia relief restored immunity.	[18]
Gastric cancer	BGC-823, HGC-27, SGC-7901 cells; xenografts	CD73	1% O_2_ or CoCl_2_	HIF-1α-mediated CD73 induction promoted adenosine production and the Warburg effect, enhancing tumour growth.	[19]

Notes: HIF-1α = hypoxia-inducible factor-1α; MDSCs = myeloid-derived suppressor cells; ADA = adenosine deaminase; NPP1 = ectonucleotide pyrophosphatase/phosphodiesterase 1; TM-PAP = transmembrane prostatic acid phosphatase; PKC = protein kinase C; TNBC = triple-negative breast cancer.

## Data Availability

No new data were generated or analysed in this study. All data discussed are available within the cited literature.

## Abbreviations

The following abbreviations are used in this manuscript:

ADA	Adenosine deaminase
ADP	Adenosine diphosphate
ALP	Alkaline phosphatase
AMP	Adenosine monophosphate
APCP	Aminophenylmethyl diphosphonate (CD73 inhibitor)
ATP	Adenosine triphosphate
BALB/c	Mouse strain used for orthotopic transplantation
BT-474	Human luminal A breast cancer cell line
BGC-823	Human gastric carcinoma cell line
CD	Cluster of differentiation
CD39	Ectonucleoside triphosphate diphosphohydrolase 1 (ENTPD1)
CD73	Ecto-5′-nucleotidase (E-5NT; NT5E)
CoCl_2_	Cobalt(II) chloride (chemical hypoxia inducer)
EMT	Epithelial–mesenchymal transition
ENTPDase	Ectonucleoside triphosphate diphosphohydrolase
E-NPP	Ectonucleotide pyrophosphatase/phosphodiesterase
E-5NT	Ecto-5′-nucleotidase
H_2_O_2_	Hydrogen peroxide
HCC	Hepatocellular carcinoma
HGC-27	Human gastric carcinoma cell line
HIF-1α	Hypoxia-inducible factor-1α
LDHA	Lactate dehydrogenase A
MCF-7	Human luminal A breast cancer cell line
MDA-MB-231	Human triple-negative breast cancer cell line
MDSC	Myeloid-derived suppressor cell
mCRC	Metastatic colorectal cancer
MeoE_2_	2-Methoxyestradiol (HIF-1α inhibitor)
NDP	Nucleoside diphosphate
NMP	Nucleoside monophosphate
NPP1	Ectonucleotide pyrophosphatase/phosphodiesterase 1
NSCLC	Non-small cell lung cancer
NTP	Nucleoside triphosphate
O_2_	Molecular oxygen
pNPP	p-Nitrophenylphosphate
Pi	Inorganic phosphate
PKC	Protein kinase C
PPi	Inorganic pyrophosphate
ROS	Reactive oxygen species
SGC-7901	Human gastric carcinoma cell line
SNP	Single-nucleotide polymorphism
TME	Tumour microenvironment
TM-PAP	Transmembrane prostatic acid phosphatase
TNBC	Triple-negative breast cancer
VEGF	Vascular endothelial growth factor
4T1	Murine mammary carcinoma cell line
